# Metabolic syndrome and its associated risk factors in Iranian adults: A systematic review

**Published:** 2015

**Authors:** Karimollah Hajian-Tilaki

**Affiliations:** Department of Biostatistics and Epidemiology, Babol University of Medical Sciences, Babol, Iran.

**Keywords:** Metabolic syndrome, cardio metabolic risk factors, adults, Iran

## Abstract

**Background:**

Metabolic syndrome (MetS) is a complex clustering cardiovascular risk factors such as abdominal obesity, hypertension, diabetes and dylipedemia. It has been a growing health problem in Iranian adults in recent decade. The objective of this article was to review the prevalence of MetS and the corresponding risk factors among Iranian adults.

**Methods:**

We conducted a systematic review to extract the published articles regarding metabolic syndrome and its risk factors among Iranian adults aged >19 years by searching in PubMed, Google Scholar, SID, Magiran and Iranmedex databases. The forty-three published articles were selected regarding MetS among Iranian adults in this review during 2005-2014.

**Results:**

From the 43 studies, the rate of MetS varied from 10% to 60% depending on sex, age and region. The highest rate reported among postmenopausal women in Shiraz was over 60%. There was almost a consistent finding that the rate of MetS was higher among women compared with men across national level except in one study. A very sharp difference (43.3% vs. 17.1%) was observed in western Iran (Kordestan province) between sexes. MetS was significantly more prevalent among older adults, postmenopausal women, less-educated people, those living in urban areas and those with low physical activity and unhealthy eating habits across national level consistently.

**Conclusion:**

An emerging high rate of MetS across national level highlights the lifestyle modification as preventive measures in Iranian population by focusing primarily on high risk profiles such as low socioeconomic background, low level of education, older age and postmenopausal women.

Metabolic syndrome (MetS), a cluster of cardiovascular risk factors is a matter of concern in both the developed and the developing countries (1, 2). It is a worldwide growing health problem. It is well established that MetS is linked with several cardiovascular events (3-6). In the United States, about aquarter of adult population suffers from MetS (7). However, this is more growing in the stage of epidemiologic transition of diseases in the developing countries such as the Middle East countries particularly, Iran (8-13). MetS was first described as x syndrome by Reaven in 1988 (14). It has four essential components including obesity/abdominal obesity, hyperlipedemia, hypertension and diabetes (2). However, so far several definitions have been proposed (1). 

The first was the World Health Organization (WHO), second the International Disease Federation (IDF), third the National Cholesterol Education Program (NCEP), and the fourth was the modified definition of the NCEP/ATP III (ATP III/ American Heart Association (AHA)/ National Heart, Lung and Blood Institute (NHLBI)) (1, 2, 15). From these, the report of the third panel of NCEP called Adult Treatment Panel (ATP III) was used widely as a common definition in the literature (1). 

ATP III provided a practical simple screening tool of diagnosis of MetS as the presence of three or more of the five criteria-emphasis on high waist circumference (WC>102 in men and WC>88 in women), high blood pressure (BP>130/80), high triglyceride (TG>150), high glucose (FBG>110, and low HDL (HDL<40 in men and HDL<50 in women) (2). On the other hand, abdominal obesity as defined by high WC is as a compulsory criterion plus the two or more of the other four criteria in IDF definition.

However, the waist circumference (WC) was recommended as a simple screening tool for measuring abdominal obesity in contrast to body mass index or other anthropometric measures. The cutoff value proposed by ATP III for WC was debate topic. This mainly may depend on ethnicity and gender (1, 2). Several studies in Asian population particularly in China, Turkey and Iran, the regional cutoff value for WC proposed might have been more appropriate (16-18). The Iranian National Committee of Obesity (INCO) also proposed a revised ATP III criteria with regional cutoff value of WC>95 cm for men and women (18). Iranian population has had an experience of demographic and epidemiologic transition stage in the recent decade. Thus, the rate of cardiovascular diseases and its mortality has increased dramatically (19-23). Changing lifestyles toward modernization and urbanization corresponds with the increasing rate of obesity and abdominal obesity as major contributors to MetS in adults and adolescents as well (21, 24, 25, 26). The relevant issues and the corresponding consequences were raised by several studies among Iranian populations (11, 12, 20, 24). Thus, the objective of this article was to review the prevalence of MetS and the associated risk factors in Iranian published studies among the adult population.

## Methods

We have reviewed the status of MetS, the prevalence and the risk factors among Iranian adults in published papers from 2005 to 2014 that mainly influenced the increasing rate of cardiovascular events in Iranian population. PubMed, SID, Google scholar, Iranmedex and Magiran data bases were used to search articles published concerning MetS in Iranian adults. We searched all these data bases for related to Iranian studies using keywords: metabolic syndrome, prevalence, adults, Iran and risk factors such demographic characteristics, lifestyle related factors including physical activity and habitual foods. Overall, 43 related articles were reviewed. 

The results were summarized with respect to sample size, type of study, region (province), the definition used for MetS, and the prevalence and the risk factors were explored. From these studies, 34 articles primarily focused on the prevalence and their sociodemographic determinants and 9 studies had a prior hypothesis and used a particular analytic design as comparative study to examine the hypothesis. Hopefully, the published studies of MetS among adults spread across the other regions (from north to south and west to east).

## Results

**Prevalence of Mets in adults:** Table 1 shows the characteristics of published studies and the corresponding prevalence of MetS in Iranian adults. From the 43 studies that we reviewed, the rate of MetS varied from 10% to 60% depending on sex, age and region. The highest rate (60.2%) was reported among the postmenopausal women in Shiraz (27). The lowest rate (22.5%) was by Sarafzadegan in Esfahan heart health study, the central part of Iran (28), Sharifi et al. in the province of Zanjan (29), the west of Iran (23.7%), in Ahvaz (22.8%) (30) and Kaykhaei in Zahedan (31), and the south of Iran, 21.0% (24.9% women vs. 15.4% men). In a health center-based study by Mahjob et al. in Babol, surprisingly, a very low rate of Mets was reported as 9.4% in males but 28.4% in females (32). However, it is not in accordance with the findings of Hajian-Tilaki et al in a population-based study in Babol. A recent study has reported by Hajian-Tilaki et al. in Babol, that the rate of MetS was 42.3% (36.5% in men and 47.3% in women) based on ATP III definition (12). The rate in Tehran adult population was 33.2% by ATP III definition and 33.2% according to IDF criteria but in another report in Tehran population among the aged >65 years, the rate was 50.8% by ATP III definition (33). Also, another report from northwest Iran (Khorasan province) the rate was 39.9% (29.1% males, 50.4% females) (34). The other report from the west of Iran, in the province of Kordestan, the rate of Mets was 29.1% (ATP III) (41.3% women vs. 17.1% men) (35). A national study of 30 provinces of Iran reported by Delavar et al. the rate of MetS was 34.7% by ATP III definition and 37.4% by IDF criteria (22). There is almost a consistent finding that the rate of MetS was higher among women compared with men across the national level. A very sharp difference (43.3% women vs. 17.1% men) was observed in western Iran (Kordestan province) between sexes (35). Only one study reported by Jamshidi in Hamedan in which a higher rate of MetS was observed in men compared with women (25.6% vs. 19.2%) (36). A very low rate of MetS among women aged 15-49 years was reported by Ebrahimi et al. in Shareza, province of Esfahan, as 9.7% and 17.3% by ATP III and IDF definitions, respectively (37). In contrast, the rate was very high in a study by Ebrahimi-Mamghani et al. among the male fire-fighters and male clerks at fire station of Tabriz as 56.6% and 60.3%, respectively (38). Moreover, a large difference in the prevalence of Mets was observed between the north and the south of Iran (12, 30, 31).

**Risk Factors of MetS**

**Demographic Factors: ** Gender: As we already noted, a clear pattern of gender relationship with Mets was present in Iranian studies (39-55). The rate is almost higher in women compared with men; singularly a much higher rate was revealed among postmenopausal women. In some studies, the rate among women had almost doubled compared with men (27, 30, 31, 33, 34, 35, 39, 43, 46).

**Aging:** The prevalence of Mets and its major component that increased with aging in all studies were reviewed. Specifically, the rate was elevated substantially in aged >50 years. In a study of Isfahan Health Heart Program, the rate of Mets among elderly aged 60 or older was 49.7% versus 17.5% aged <60 years (46). Also, an emerging high rate of Mets and its major component was reported at age 50 or older by Hajian-Tilaki et al. in a study of adult population living in urban areas in northern Iran and all major components of MetS had increased with aging (12).

**Socioeconomic status:** There is an unequal variation in MetS according to socioeconomic status and in Iranian adults. It was shown that Mets is more prevalent in low socioeconomic status particularly the low-educated adults compared with the highly-educated ones (12, 24, 56). Several studies have shown that the components of MetS such as obesity/central obesity and diabetes are more common in the illiterate and low educated subjects (12, 24 56). 

**Lifestyles Risk Factors**

**Obesity/Abdominal Obesity: **Several lifestyle-related risk factors for MetS were documented in different studies (57-71). There is a strong interrelation between obesity, central obesity with hypertension, diabetes, hyperlipedemia and MetS. It has been clearly established the obesity/central obesity is the central component of Mets (1, 2, 14). Obesity as measured by body mass index(BMI) and abdominal obesity by waist circumference (WC) or waist to hip ratio(WHR) or waist to height ratio (WHtR) are the prognostic predictors of diabetes, hypertension, hyperlipedimia and thus the occurrence of Mets (16-23). Notably both obesity and abdominal obesity have a discriminatory ability to predict non-obese components of MetS in Iranian adults (23). 

 A high rate of obesity and central obesity has been reported in two recent decades in Iranian adults (24, 43, 45, 49). The rate was almost higher in women compared with men (22). Principally, a higher rate of abdominal obesity was reported in Iranian women that lived in the urban areas in the north of Iran (24). A study compared Mets in a sample of 1194 Iranian adults versus 1386 French adults. The rate of Mets in Iranian women was 55% versus 13.7% in French women and 30% Iranian men versus 13.7% French men (43). High rates of HP (48%), TG (42.8%) and low HD (81.8%) were observed in Iranian men while the high rate of central obesity (65%), HP (52.1%), high TG (43.15%) and low HDL (92.7%) had been revealed in Iranian women (43). 

 In another study, in south of Iran (31), although a relative lower rate (22.8%) of Mets was reported in adults but the rate of major components of Mets alone was relatively high (abdominal obesity 29.4%, high TG 40.2%, low HDL 40.2%, HP 15.4% and high FBS 37.8%). A sharp difference with higher rate of MetS was revealed in the north of Iran compared to south (22, 31). A study among Iranian professional drivers, 41.4% were overweight and 21.3% were obese (44). A population-based study of adults in the north of Iran 10% and 30% of adults were obese in men and women, respectively while the rate of abdominal obesity was much higher mainly among women compared with men (46% versus 18%) (24). A report from south of Iran, although showed a relative lower rate of MetS compared to the north, but the low HDL (60.6%), high WC (43.3%) were the most common components of Mets followed by high TG (32.0%, high FBS (17.1%) and high BP (13.0%) (31). In a study in Turkaman ethnic women, a much higher rate of different components of Mets was observed. The high WC (75%) and low HDL (70.6%) were the most common components followed by high TG (35.5%), high FBS (29.4%) and high BP (26.2%) (49).

**Physical activity and Mets: **Among all possible lifestyle- related risk factors, a clear link has been revealed between physical activity and Mets, and obesity and central obesity (24). Primarily, the physical activity at vigorous level decreased the risk of Mets substantially compared with low active but not the moderate level (12). Also, a dose response has revealed the relationship of physical activity and Mets, obesity and abdominal obesity both among children and adolescents as well (25, 26). In Tehran Lipid and Glucose study, the prevalence of Mets was higher among the obese group (58.2%) compared with overweight (36.6%) and normal weight (18.1%).The normal weight subjects were more physically active than other groups (58). The increased level of leisure-time physical activity was associated with decreasing in the likelihood of abnormality in components of MetS and thus, the occurrence of Mets (58).

**Habitual foods and MetS: **Besides physical activity, the habitual Iranian foods including high consumption of rice and bread may have an important role in apparent high rate of Mets and its major components. Tehran Lipid Glucose study reported the higher BMI and higher TG with high consumption of sugar drinks (3 times per week) compared with low consumption (less than one time per week) but after adjustment the difference disappeared (59). In a case control study, the prevalence rate of MetS was lower as compared with the lowest quartile of legume intake (167% versus 46.7%) (60). After adjusting the possible confounding factors, a decrease in triglyceride concentration, fasting blood sugar, systolic blood pressure and increase in HDL-C concentration were observed by quartile categories of legume intakes (60). Thus, dietary legume intake is inversely associated with MetS. In addition, in another study of Tehranian population, the prevalence of MetS was higher among subjects in lower quartile of fruits (17.2% in the first quartile versus 15.4% in the 4th quartile) (61). Those with higher quartile of vegetable intake had lower risk of MetS. A significant difference of the mean of vegetable and fruit intake was observed between subjects with and without MetS (61). A similar finding also was reported by another cross-sectional study among Iranian teachers aged 40-60 years (62). 

Thus, fruit and vegetable intakes were inversely associated with the risk of MetS. In addition, in a population-based cross-sectional study of Tehranian adults, dietary calcium levels and dietary vitamin D level were inversely associated with MetS after adjusting several potential confounding factors (63). 

A significant difference in the prevalence of MetS was found between the first and fourth quartiles of dietary calcium intake and vitamin D levels (63). However, for a net clarification of these associations, the prospective studies are needed to investigate the influence of habitual food patterns on the risk of MetS among Iranian adults. 

**Table 1:** Characteristics of published studies and the prevalence of MetS among Iranian adults

**Authors**	**Sample Size**	**Type of Study**	**Province**	**Criteria**	**Prevalence of Mets**
Zabetian et al. ^(^^33^^)^ 2007	10368 men & women aged≥20yr	Population based Cross- sectional	Tehran (Lipid Glucose Study)	IDF ATPIII WHO	32.1% (95%CI: 31.2-3.0%) 33.2% (95%CI: 32.3-9.1%) 18.4%(95%CI:17.6-19.2)
Sadbafoghi et al.^( ^^55^^)^ 2007	1110 men & women aged 20-74 yr	Population based Cross- sectional	Yazd (urban)	ATP III	32.1%
Azimi Nezhad etal. ^(^34^)^ 2008	2353 men & women aged 15-64 yr	Population based Cross- sectional	Khorasan province (urban & rural area)	IDF ATP III AHA	40.5% (26.0% men vs. 54.5% women) 39.9% (29.1% men vs. 50.4% women) 40.5% (30% men vs. 50.15 women)
Fakhrzadeh et al. ^(^^39^^)^ 2006	1573 men & women aged 20-64 yr	Clinic based (Laboratories of Tehran Uni) Cross-sectional	Tehran	ATP III	27.5% age adjusted (20.3% men vs. 35.9% women)
Hadegh et al. ^(^^40^^)^ 2009	720 men & women aged >65 yr	Population based Cross- sectional	Tehran (Lipid Glucose Study)	IDF ATP III WHO	41.9% 50.8% 41.8%
Esteghamati et al. ^(^^41^^)^ 2009	3296 men & women	Population based Cross- sectional	Representative samples of Iranian adults	IDF	24% to 30% depending on sex
Jalali et al. ^(^^51^^)^ 2009	1402 men & women aged 19-90 yr	Population based Cross- sectional	Fars (rural area)	ATP III IDF	29.0% 30.5% Female to male ratio=1.97
Shanfi et al.^(^^29^^)^ 2009	2941 men & women aged≥20 yr	Cross-sectional	Province of Zanjan (urban)	ATP III	23.7% (95%CI: 22.0-25.0%) 23.1% men vs. 24.4% women
Saberi et al. ^(^^42^^)^ 2011	429 bus and truck drivers (men)	Cross-sectional	Kashan	ATP III	35.9%
Sarafrazadeghan et al^(^^28^^)^ 2011	9570 men & women aged≥19yr	Population based Cross- sectional	Isfahan Healthy Heart Program	ATP III	22.5%
Azimi-Nikzad et al. ^(^^43^^)^ 2012	1194 Iranian adult and 1386 French adults (men & women)	Population based Cross- sectional	Iranian adults vs. French adults	ATP III	55% Iranian women 30% Iranian men vs. 13.7% French men 6.6% French women
Sharrafzadegan et al. ^(^^46^^)^ 2012	9572 (men& women) aged ≥19 yr	Population based Cross-sectional	Isfahan Healthy Heart Program	ATP III	49.5% aged ≥60 yr vs. 17.5% <60 yr. aged ≥ 60 yr: 59.2% women vs. 39.8% men
Kaykhaei et al.^(^^31^^)^ 2012	1802 (735 men & 1067 women) aged ≥19 yr	Population based Cross-sectional	Zahedan (South of Iran)	ATP III IDF AHA	21.0% (14.4% men vs. 24.9% women) 24.8% (20.0% men vs. 28.1% women) 23.3% (19.7% men vs. 25.8% women)
Esmailnasab et al.^(^35^)^ 2012	1194 men & women aged 25-54 yr	Population based Cross-sectional	Kurdistan (West of Iran)	ATP III	29.1% (17.1% men vs. 41.3% women)
Raheb Gorbani et al. ^(^^53^^)^ 2012	3799 men & women aged 30-70 yr	Population based Cross- sectional	Semnan (rural & urban)	ATP III IDF	28.5% 35.8%
Mohebbi et al. ^(^^44^^)^ 2012	12138 men aged 20-67 yr	Professional drivers cross-sectional	West Azerbaijan	IDF	Age adjusted 32.4%
Mahjoub et al. ^(^^38^^)^ 2013	933 men & women aged≥18 yr	Health center based Cross- sectional	Mazandaran, Babol	ATP III INCO	23.7% (95%CI: 21.0-26.4%) (9.4% men vs. 28.4% women) 15.7% men vs. 22.5% women
Jouvandeh et al. ^(^^45^^)^ 2013	118 post menopausal women	Clinic based cross-sectional	Tehran Menopause Clinic	ATP III	30.1%
Shabazian et al. ^(^^30^^)^ 2013	912 men & women Mean aged 42.3±14 yr	Population based cross-sectional	Ahvaz (South west of Iran)	ATP III	22.8% (15.9% men vs. 29.1% women)
Maharouei et al. ^(^^27^^)^ 2013	490 Pre-menopausal women & 434 postmenopausal	Cross-sectional	Shiraz	ATP III IDF	60% post menopausal 59.4% postmenopausal
Esmailzadeh et al. ^(^^35^^)^ 2013	529 men & 578 women aged 20-75 yr	Population based Cross-sectional	Gazvin	WHO ATP III ADF	28.0% 26.2% 34.2%
Marjani et al. ^(^^48^^)^ 2012	100 postmenopausal women	Clinic based Cross sectional	Gorgan	ATP III	31.0%
Shahini et al. ^(^^49^^)^ 2013	160 women Mean aged 32.3±13.7 yr	Cross-sectional	Gorgan (Turkmen ethnic)	ATP III	35%
Hajian-Tilaki et al. ^(^^12^^)^ 2014	1000 men & women aged 20-70 yr	Population based Cross-sectional	Mazandaran, Babol (urban)	ATP III	42.3% (36.5% men vs. 47.3% women)
Javadi et al. ^(^^52^^)^ 2014	996 men & women aged ≥24 yr	Population based Cross- sectional	Gazvin	ATP III	33.0% (30.9% men vs. 39.9% women)
Ebrahimi et al. ^(^^37^^)^ 2008	1501 women aged 15-49 yr	Population based cross sectional	Shareza (rural & urban)	IDF ATP III	17.3% 9.7%
Jamshidi et al. ^(^^36^^)^ 2014	550 men & women aged 40-80 yr	Clinic based Cross sectional	Hamadan	ATP III	25.6% (men vs. 19.2% women)
Ebrahimi-Mamghani et al.^(^^38^^)^ 2011	76 fire fighting men & office worker men	Cross sectional	Tabriz	ATP III	56.6% fire fighting men vs. 60.3% office worker
Delavari et al. ^(^^32^^)^ 2009	3024 men & women aged 25-64 yr	Population based cross sectional	National level 30 provinces	ATP III IDF	34.7% (95% CI: 33.1-36.3%) 37.4% (95% CI: 35.9-39.0%)
Ostovareh et al. ^(^^54^^)^ 2014	5826 adults (Amole) 2243 adults (Zahedan)	Population based cross sectional	Amole & Zahedan	ATP III IDF	27.6% Amole vs. 12.0% Zahedan 26.9% Amole vs. 11.8% Zahedan
Sarrafzadegan et al.^(^^68^^)^ 2008	12514 adults men & women aged ≥ 19	Population based cross sectional	Esfahan, Najaf-Abad, Arak (rural & urban, Isfahan Healthy Heart Program)	ATP III	23.3% (35.1% women vs. 10.7% men) 24.2% urban vs. 19.5% rural
Delavar et al. ^(^^54^^)^ 2009	984 women aged 30-50 yr	Clinic based cross sectional	Babol (urban)	ATP III	31.0%
Malek et al. ^(^^66^^)^ 2014	800 men & women aged ≥35	Population based cross sectional	Borujerd (West of Iran)	ATP III	43%
Heidari et al. ^(^^67^^) ^ 2010	1596 women aged >45 yr	Population based cross sectional	Esfahan, Najaf-Abad, Arak (Isfahan Healthy Heart Program)	ATP III	44.9% premenopausal 57.9% early menopausal 64.3% postmenopausal
Noori et al. ^(^^63^^)^ 2007	808 men & women Aged 18-74	Population based cross sectional: comparative study	Tehranian adults	ATP III	Calcium & Vitamin D intakes↓ MetS
Esmailzadeh et al. ^(^^61^^) ^ 2007	486 women aged 40-60	cross sectional	Tehranian Teachers	ATP III	vegetables & fruits↓ MetS
Falahi et al. ^(^^62^^)^ 2013	973 men & women	Cross sectional	Korram-Abad city	ATP III	Healthy dietary intake↓ MetS
Tabesh et al. ^(^^59^^)^ 2011	1752 men & women (adults)	Cross sectional	Najaf-Abad & Arak	ATP III	Sugared drinks not associated with MetS
Bahaderian et al. ^(^^69^^)^ 2014	Mean age 37.8± 12.3 yr	Prospective	Tehranain adults (Tehran Lipid Glucose Study)	ATP III	Unhealthy snakes ↑MetS
Shab-Baldar et al. ^(^^71^^) ^2014	2750 men & women aged 20-74 yr	Population based Cross-sectional	Tehranian adults (Tehran Lipid Glucose Study)	ATPIII	Fatty acid consumption (poly saturated and unsaturated fatty acid intake) ↑MetS
Hoseini et al. ^(^^70^^)^ 2007	606 men & women Aged 18-74 yr	Population based Cross-sectional	Tehranian adults (Tehran Lipid Glucose Study)	ATP III	Fruits & vegetables consumption↓ MetS
Fam et al. ^(^^58^^) ^ 2012	4665 men & women aged 20-70 yr	Population based Cross-sectional	Tehranian adults (Tehran Lipid Glucose Study)	ATP III	Increased level of Leisure time physical activity ↓ the risk of inappropriate changes in components of MetS and the occurrence of MetS
Hoseinpor-Niazi et al. ^(^^60^^)^ 2011	70 MetS and 160 healthy subjects aged 25-55 yr	Case-control	Tehranain adults	ATP III	Dietary legume intakes↓ MetS

## Discussion

Our systematic review showed that the rate of MetS ranged from 10% to 60% among Iranian adults depending on the age, gender and region. The prevalence was more common among women, older age, and low socioeconomic status. Specifically, it was more prevalent in low-educated and inactive adults. A very high prevalence rate of major components of MetS such as obesity, central obesity, hyperlipidemia (high TG and High LDL), high BP and high FBS had been reported consistently across national level.

Although, a few studies reported the prevalence of MetS about10- 20% but in the majority of studies, the rate was higher than 30% in Iranian adults. An emerging high prevalence rate was observed in population in North of Iran (12). A similar high rate of MetS has been reported among adults in Pakistan and Oman and Turkish population as well (9, 17, 64). The range of MetS in our neighborhood countries varied from 32-47% in women and 20-37.2% in men (65). This is rather similar to the findings of the studies that we reviewed while the rate of MetS is more prevalent in Iranian adults than many other populations in Europe, US, Latin American, East Asia and India (7, 8, 43). In our review, a study compared the rates of MetS between Iranian adults and French adults, a sharp difference in prevalence of MetS had been observed between two populations in both genders. This difference primarily attributed to the differences in culture and lifestyles (43).

A relative large variation of the prevalence of MetS has been observed between provinces across the country especially from south to north of Iran (12, 31). Several factors may explain this difference. The lifestyles may differ between ethnic groups. The higher rate in the north may be associated with the difference in physical activity and habitual food patterns in particular the high consumption of rice among people living in the north. The difference of demographic profiles of people that were recruited in the study and the definition used as MetS may be another source of explanation of the apparent disparity across studies.

Several factors such as older age, being a female, lowsocio economic status, low education and low physical activity were reported to be associated with MetS (12). These risk factors are primarily associated with obesity, in particular, abdominal obesity as well (24, 56). A similar finding also reported from other populations (9, 17). The higher rate of MetS in Iranian women are attributed to the greater rate of abdominal obesity because of lower physical activity level, higher order of live births, estrogen receptor and going – through menopause (24). Central obesity as measured by waist circumference or waist to height ratio as a simple tool for diagnosis has a greater discriminatory performance for screening non-obese components of MetS such as diabetes, hypertension and dyslipedemia (23). Iranian habitual diet such as high consumption of rice and white bread and sweets and lower intake of vegetable and fruits and legume has a major contributory role on the pattern of abdominal obesity and thus MetS occurs in Iranian population (59-63). 

It has been shown that adults with low socio economic status and low education have greater risk of cardio metabolic risk factors (12) since they are more inactive and may use more unhealthy habitual foods because of lack of health consciousness, unawareness, having negative attitude and limited financial resources. In addition, the modern lifestyles such as fastfoods with high calorie intakes play a contributor role on the components of MetS in particular on obesity and central obesity. On the other hand, the healthy dietary intakes such as fruits and vegetables and dietaries with high level of calcium and vitamin D were related to the lower risk of triglyceride, diabetes, hypertension and MetS (62). Some of these evidence was demonstrated in cross- sectional and case-control studies among Tehranian adults (61-63, 69-71). However, more prospective studies are needed to establish a clear evidence for an interventional plan in the public health management as preventive measures.

In conclusion, the prevalence of Mets in Iranian adults is higher than western counterparts. An emerging high prevalence of MetS in Iranian adults, highlights an urgent population based interventional plan to cope with modern lifestyles and to replace healthy lifestyles as preventive measures by focusing on high risk profile such as low socio economic, low level of education, older age and being women.
